# Experimental infection with the *Toxoplasma gondii* ME-49 strain in the Brazilian BR-1 mini pig is a suitable animal model for human toxoplasmosis

**DOI:** 10.1590/0074-02760140318

**Published:** 2015-02

**Authors:** Farlen José Bebber Miranda, Diogo Benchimol de Souza, Edwards Frazão-Teixeira, Fábio Conceição de Oliveira, João Cardoso de Melo, Carlos Magno Anselmo Mariano, Antonio Peixoto Albernaz, Eulógio Carlos Queiróz de Carvalho, Francisco Carlos Rodrigues de Oliveira, Wanderley de Souza, Renato Augusto DaMatta

**Affiliations:** 1 Laboratório de Biologia Celular e Tecidual; 2 Unidade de Pesquisa Urogenital, Universidade do Estado do Rio de Janeiro, Rio de Janeiro, RJ, Brasil; 3 Laboratório de Sanidade Animal; 4 Faculdade de Medicina de Campos, Campos dos Goytacazes, RJ, Brasil; 5 Laboratório de Clínica e Cirurgia Animal; 6 Laboratório de Morfologia e Patologia Animal, Universidade Estadual do Norte Fluminense Darcy Ribeiro, Campos dos Goytacazes, RJ, Brasil; 7 Laboratório de Ultraestrutura Celular Hertha Meyer, Universidade Federal do Rio de Janeiro, Rio de Janeiro, RJ, Brasil

**Keywords:** Toxoplasma gondii, pig infection, haematology, parasitaemia, neutrophils

## Abstract

Toxoplasma gondii causes toxoplasmosis, a worldwide disease. Experimentation with pigs is necessary for the development of new therapeutic approaches to human diseases. BR-1 mini pigs were intramuscularly infected with T. gondii with tachyzoites (RH strain) or orally infected with cysts (ME-49 strain). Haematology and serum biochemistry were analysed and buffy coat cells were inoculated in mice to determine tachyzoite circulation. No alterations were observed in erythrocyte and platelet values; however, band neutrophils increased seven days after infection with ME-49. Serology of the mice inoculated with pig blood leucocytes revealed circulating ME-49 or RH strain tachyzoites in the pigs' peripheral blood at two and seven or nine days post-infection. The tachyzoites were also directly observed in blood smears from the infected pigs outside and inside leucocytes for longer periods. Alanine-aminotransferase was high at days 21 and 32 in the RH infected pigs. After 90 days, the pigs were euthanised and their tissue samples were processed and inoculated into mice. The mice serology revealed the presence of parasites in the hearts, ileums and mesenteric lymph nodes of the pigs. Additionally, cysts in the mice were only observed after pig heart tissue inoculation. The infected pigs presented similar human outcomes with relatively low pathogenicity and the BR-1 mini pig model infected with ME-49 is suitable to monitor experimental toxoplasmosis.

*Toxoplasma gondii* causes toxoplasmosis, a worldwide disease that infects one third of the human population (Flegr & Stříž 2011). Swine used in research is usually of the *Sus scrofa domesticus* species, the farm or the miniature breed. The swine miniature breeds most frequently used in research are the Yucatan, Hanford, Göttingen and Sinclair Hormel breeds (Swindle et al. 1994). However, other miniature breeds exist, such as the Brazilian miniature pig, BR-1, which was developed exclusively for biomedical experimentation (Mariano 2003); however, this breed has never been reportedly used as a *T. gondii* pig model. Miniature pigs have been used as a biomedical model due to their physiological similarities to man, including neonatal development, blood flow, host defence (Brown & Terris 1996) and infectious disease outcomes (Groenen et al. 2012). Because of these similarities, the relevant knowledge obtained with lower mammals should be validated in pigs. Thus, swine constitute an interesting animal model for preclinical experimentation (Vodicka et al. 2005). Additionally, pig meat is one of the most important sources for *T. gondii* cyst ingestion by humans (Dubey 1986 ). To endorse the BR-1 pig *T. gondii* model, infections were performed and clinical signs were registered and haematological and bioassays in mice were conducted. Moreover, in view of recent observations regarding nephron loss as well as light glomerular and tubular lesions in experimentally infected mice, a similar study was carried out in pigs.

## MATERIALS AND METHODS

*Animals and parasitic strains - *Twelve castrated female BR-1 mini pigs, eight months of age were used in this study. These animals were acquired from Mini Pig Research and Development, Campina do Monte Alegre, state of São Paulo, Brazil (revistapesquisa.fapesp.br/en/2006/09/01/mini-pigs-in-the-laboratory/). These animals were delivered to the Darcy Ribeiro North Fluminense State University (UENF), state of Rio de Janeiro, Brazil, and were maintained in three randomly assigned groups of four individuals at a medium size animal facility, with a controlled temperature (25ºC) as well as food and water *ad libitum* for two weeks before the experimental procedures.

Swiss Webster mice were bred at the UENF mouse animal facility and were infected separately with the RH and ME-49 *T. gondii* strains, which carry high and low mortality in mice, respectively. The RH strain was maintained by intraperitoneal injections of tachyzoites and the ME-49 strain was maintained by inoculation of tissue cysts. The cysts were obtained from the brains of the mice three months after infection. The brains were removed, washed with phosphate buffered saline (PBS), macerated and homogenised. After quantification, the tachyzoites or cysts from the mice were used to infect the pigs.

*Ethics *- This study was carried out in strict accordance with the Brazilian Law #11794/08. The animal study protocol was reviewed and approved by the UENF Committee on the Ethics of Animal Experiments (permit 130).

*Infection, blood collection and fundoscopy - *Two groups of four pigs each were infected and a third group was a non-infected control group. The "ME-49" group was infected by an oral route with 660 cysts of ME-49 strain and the "RH" group was infected intramuscularly with 1 x 10^7^ RH strain tachyzoites, which is a non-cystogenic strain. After infection, the clinical signs of the animals were observed daily. The pig infections were confirmed by serological testing with the modified agglutination test (MAT) (Dubey & Desmonts 1987) before and 30 days after infection. The pigs were sedated with an intramuscular acepromazine (0.22 mg/kg) injection that was combined with ketamine (20 mg/kg) for blood collection and fundoscopy of all animals. The blood was collected from the auricular vein with ethylenediamine tetracetic acid (EDTA) as an anti-coagulant and purified for serum obtainment. Both procedures were performed three times a week in the first 14 days, twice a week for an extra 16 days and weekly up to 90 days. Indirect fundoscopy was performed with an ophthalmoscope (ODN 4.4; Eyetec).

*Haematology and serum biochemistry *- Blood samples (3-5 mL) with EDTA from all of the animals were processed for automatic white blood cell, red blood cell and platelets counting (MS4 Melet Schloesing^(r))^. Haemoglobin concentrations, haematocrit percentages, the mean corpuscular volumes and the mean corpuscular haemoglobin concentrations were also assayed by a cited automated procedure. In parallel, blood smears were stained with Giemsa in order to identify and estimate the differential leucocytes counts and to detect possible morphological alterations and the presence of parasites. One hundred leucocytes were differentially counted per animal.

Serum was collected from 5 mL of blood right after coagulation, stored in individual vials and frozen (-70ºC) until biochemistry determination of the following was conducted: urea, creatinine, alanine aminotransferase (ALT), aspartate aminotransferase and alkaline phosphatase. The serum biochemistry was assayed in a semi automatic spectrophotometer (BTS 310 BioSystems^(r))^.

*Parasitaemic determination *- During the first 14 days, parasitaemia was determined by mouse bioassays. Blood collected with EDTA (5 mL) was centrifuged (500 *g*, 10 min), the buffy coat was collected and washed once with PBS and the cell pellet was resuspended in 1.5 mL of PBS. This suspension was inoculated intraperitoneally (0.5 mL) in three adult mice. After 40 days, the serum of the mice that survived was submitted to the MAT with a cut-off titre of 1:25 (RH and ME-49 groups) and the brains were observed for the presence of cysts (the ME-49 group only).

*Necropsy and histopathology* *-* After 90 days post-infection (dpi), all of the pigs were euthanised, necropsied and the tissues was processed for pathology examination. Tissues were fixed in 10% buffered formalin, routinely processed in alcohol and xylol, embedded in paraffin and 5 μm sections were stained with haematoxylin and eosin. Eleven organs were analysed, including the heart, lung, liver, spleen, kidney, ileum, brain, mesenteric lymph node, skeletal muscle, tongue and retina.

*Renal stereology *- The kidney volumes of all four pigs in each group (infected with the ME-49 or RH strains and non-infected control), cleared of adipose tissue, was measured by the water displacement method. The right kidneys were transversely sliced in 2 cm sections that were fixed in 4% formaldehyde for later cortical-medullar ratio determination by the point-counting method (de Souza et al. 2011 ) and the absolute cortical volume was calculated by multiplying the cortical-medullary ratio by the renal volume. The left kidneys were sectioned frontally and then randomly picked cortical area fragments were fixed in 10% formaldehyde and routinely processed for paraffin embedding. From each animal, 26 histological fields that were obtained from different cortex sections were acquired with a digital camera that was coupled to a microscope. An M42 test-system was used for glomerular volume density estimation by the point-counting technique. The volume weighted mean glomerular volume was estimated with the point-sampled intercept method (de Souza et al. 2012) and 50 glomeruli per animal were analysed. The total glomeruli number per animal estimation was calculated by multiplying the cortical volume by the glomerular volume density and then divided by the volume weighted mean glomerular volume.

*Tissue bioassay in mice *- Eleven organs from each pig were analysed with the bioassay method and the following were collected and processed: approximately 100 g of heart, lung, liver, spleen, kidney, brain, muscle and tongue tissue, 10 cm ileum fragments, an entire eye and the mesenteric lymph nodes. Tissues were digested in pepsin and their homogenates were inoculated in mice according to the Dubey (1998) protocol, with minor modifications, as follows. Each tissue was ground separately with saline and the homogenates were incubated for 60 min in a shaker at 37ºC with 200 mL of pre-warmed acidic pepsin solution (0.52%, pH 1.2). The product was filtered through two layers of gauze and centrifuged at 1,200 *g* for 10 min. The supernatant was poured off, the sediment was resuspended in 20 mL of PBS and it was then transferred to a 50 mL centrifuge tube with a conical bottom and neutralised with 15 mL of sodium bicarbonate (1.2%, pH 8.3). After it was mixed, the solution was centrifuged at 1,200 *g* for 10 min and the sediment was resuspended with 10 mL of PBS that contained 1,000 units of penicillin and 100 μg/mL of streptomycin. Blender jars, cutting boards and other materials were sterilised with soap and hot water (100ºC) between tissue processing. One millilitre of tissue homogenate was inoculated intraperitoneally for each of three adult mice and a control group was inoculated with the same volume of PBS. Serology and brain cyst searches were performed for parasitaemia determination.

*Statistical analysis *- The numerical data were analysed statically and compared using the Wilcoxon test and the one-way-ANOVA followed by Dunnett's test. p < 0.05 was considered significant.

## RESULTS

*Clinical data *- Two pigs out of four per group became apathetic and lethargic between 2-9 dpi for the RH group and 4-11 dpi for the ME-49 group. One RH group pig, which had clinical acute phase signs of the disease, presented increased and abnormal aggressiveness during the chronic phase. No ophthalmic lesion was found in the pigs from both infected groups.

**Fig. 1:**
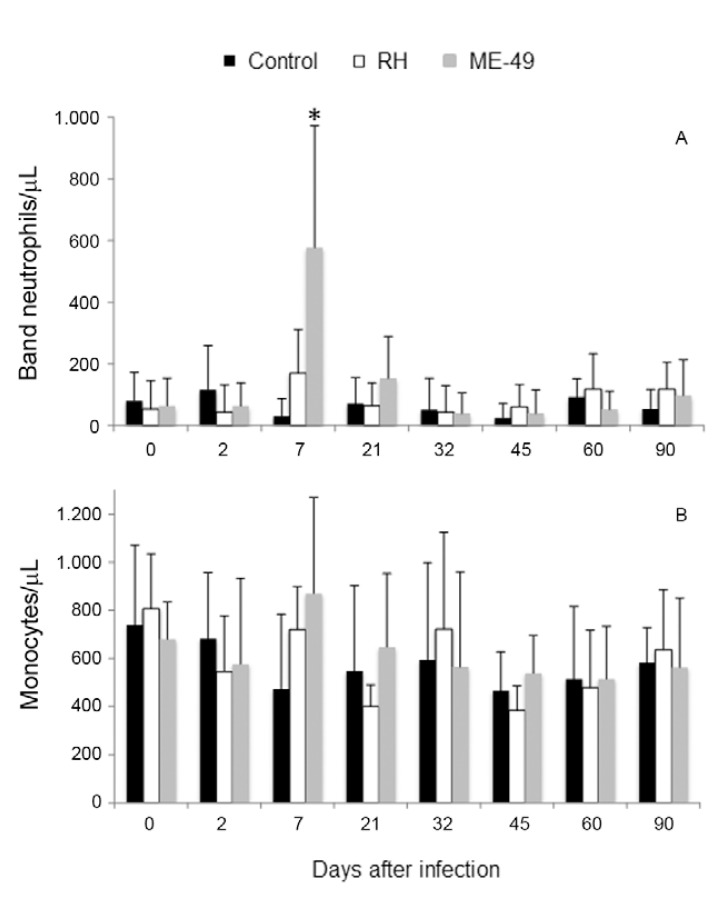
neutrophils and monocytes numbers during the infection of BR-1 pigs with Toxoplasma gondii of the ME-49 or RH strains. Band neutrophils (A) and monocytes (B) were differentiated counted in each of the four animals per group to determine possible changes caused by the infection; data are presented as means and standard deviation per group. An increase in the number of band neutrophils and a tendency for monocytosis at seven days post-infection in pigs of the ME-49 group (gray) were observed when compared with the RH (white) and control (black) groups. Asterisk means significantly different (p < 0.05) from the respective non-infected group value.

*The haematological and serum biochemistry parameters *- No alterations in the global number of leucocytes, erythrocytes or platelet values were found among the pig groups, but a significant increase in band neutrophils was seen at 7 dpi in the ME-49 group (Fig. 1A). Moreover, we observed a tendency for monocytosis at 7 dpi in the ME-49 group pigs (Fig. 1B). These changes were not seen in the RH and control group pigs.

**Fig. 2:**
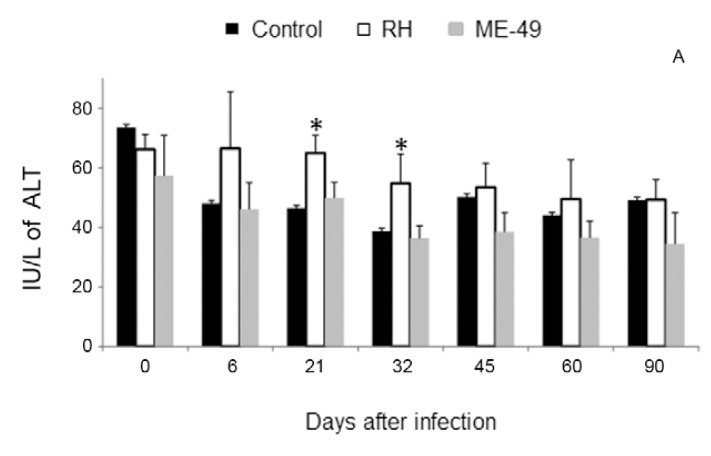
alanine aminotransferase (ALT) activity during the infection of BR-1 pigs with Toxoplasma gondii of the ME-49 or RH strains. ALT was evaluated in each of the four animals per group to determine possible changes caused by the infection; data are presented as means and standard deviation per group. A tendency at six days post-infection (dpi) and a significant increase at 21 and 32 dpi were detected when pigs were infected with the RH strain (white). The control group is disposed in black and the ME-49 in gray. Asterisk means significantly different (p < 0.05) from the respective non-infected group value.

The serum ALT biochemistry analyses in the RH group showed an increase tendency at 6 dpi, but a significant increase between 21-32 dpi was observed compared with the controls at those days (Fig. 2). All other serum biochemistry assays were not different during the infection period.

**Fig. 3:**
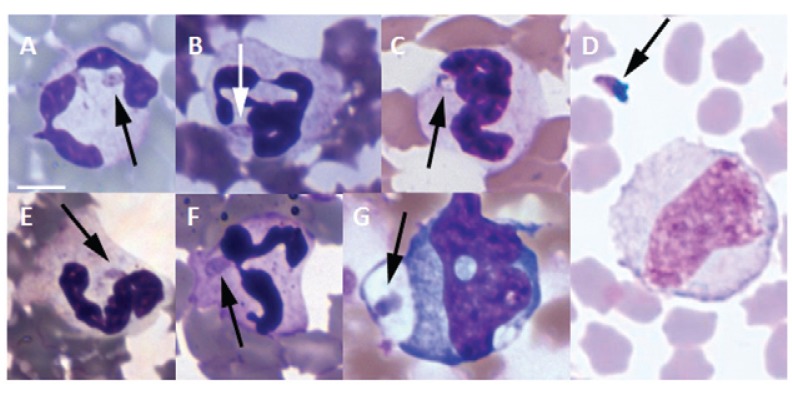
leukocytes from blood smears of pigs infected with the RH or the ME-49 strain of Toxoplasma gondii. Neutrophils infected by tachyzoites (arrow) of the RH strain at two (A), four (B) and 49 days post-infection (dpi) (C). In D, an extracellular tachyzoite of the RH strain can be seen at 63 dpi next to a monocyte. Neutrophils infected with tachyzoites of the ME-49 strain can be seen at 2 (E) and 54 (F) dpi and in a monocyte at 7 dpi (G). Bar = 5 μm.

*A bioassay in the mice that were administered swine blood leucocytes* - A bioassay test that was performed with the harvested blood leucocytes showed parasitaemia only at 9 dpi in the RH group pigs and at 2 and 7 dpi in the ME-49 group pigs (Table I). However, tachyzoites were observed in the peripheral blood of the pigs before and after these periods. The RH group pigs showed tachyzoites inside neutrophils at early (Fig. 3A, B) and late (Fig. 3C) dpis and they were also observed outside leucocytes in the late infections (Fig. 3D). Additionally, ME-49 strain tachyzoites were also seen in neutrophils at early (Fig. 3E) and late dpis (Fig. 3F) and in monocytes in the early infections (Fig. 3G). As expected, no parasites were found in the blood of the control group animals.

**TABLE I:** Bioassay in mice inoculated with blood leukocytes of pigs experimentally infected with RH or ME-49 *Toxoplasma gondii* strains

	Pig ID	ME-49					RH			
dpi^*a*^		4	5	11	12		3	1	2	10
2		+^*b*^	-	-	-		-	-	-	-
4		-	-	-	-		-	-	-	-
7		-	+	-	-		-	-	-	-
9		-	-	-	-		-	-	-	+
10		-	-	-	-		-	-	-	-
12		-	-	-	-		-	-	-	-
14		-	-	-	-		-	-	-	-

*a*: days post-infection; *b*: seropositive by the modified agglutination test (cut-off titre 1:25).

*Tissue bioassay in the mice* - Serology of the mice that received tissues from the ME-49 group pigs that were infected for 90 days indicated the presence of *T. gondii* in the heart, mesenteric lymph nodes and ileum (Table II). However, cysts were only visualised in the brains of the mice that were inoculated with the pig hearts (Table II). Inoculation of tissues from the RH group did not serum convert the mice because this strain does not form cysts in pig tissues (Dubey et al. 1994).

**TABLE II:** Bioassay in mice inoculated with tissues from pigs experimentally infected with the ME-49 strain of *Toxoplasma gondii*

Pig ID	Spleen	Heart	Brain	Mesenteric lymph node	Retina	Ileum	Skeletal musculature	Lung	Liver	Tongue	Kidney
4	-	+^*a,b*^	-	-	-	-	-	-	-	-	-
5	-	-	-	-	-	-	-	-	-	-	-
11	-	-	-	-	-	-	-	-	-	-	-
12	-	+^*a*^	-	+^*a*^	-	+^*a*^	-	-	-	-	-

*a*: seropositive by the modified agglutination test (cut-off titre 1:25); *b*: positive for cyst visualisation in mice brain tissue.

*Histological analyses* - No histopathology alteration or cysts were seen in the pig tissues examined. Additionally, the stereological analysis of the swine kidneys showed no significant differences among the groups. These numerical results are presented in Table III.

**TABLE III:** Kidney stereological data expressed as mean and standard deviation of all four pigs per group experimentally infected with strains of Toxoplasma gondii or inoculated with phosphate buffered saline (control)

Parameter	Control	RH	ME-49	p
Kidney volume (cm^3^)	51.8 ± 4.2	70.5 ± 8.0	65.0 ± 4.5	0.09
Cortical-medullary ratio (%)	16.4 ± 0.6	14.5 ± 1.2	13.4 ± 0.6	0.12
Cortical volume (cm^3^)	8.6 ± 1.3	10.0 ± 1.3	8.8 ± 1.3	0.73
Vv [Glom]^*a*^ **(%)	4.6 ± 0.4	3.5 ± 0.4	4.4 ± 0.3	0.15
VWGV^*b*^ (10^4 ^µm^3^)	102.8 ± 4.0	104.3 ± 1.7	92.0 ± 7.5	0.22
N [Glom]^*c*^ (x 10^7^)	4.0 ± 0.9	3.5 ± 0.8	4.4 ± 8.5	0.81

*a*: glomerular volume density; *b*: volume weighted mean glomerular volume; *c*: numbers of glomeruli per kidney.

## DISCUSSION

Clinical signs of toxoplasmosis were observed during the acute phase of the disease in two pigs of each group infected with the RH or the ME-49 strains. These results differ from oocysts infection (AS-28 strain) in which no major clinical alterations were observed in infected animals, except a moderate elevation of rectal temperature (Yai et al. 2003). Moreover, our clinical results are different from those reported by Wingstrand et al. (1997), in which pigs were infected with oocysts (SSI-119 strain) and cysts (SSI-119 and R92 strains). They observed severe clinical signs between 3-5 dpi, such as inappetence, transient fever and diarrhoea; however, the authors used eight-week-old pigs, which are more susceptible to *T. gondii* infection. It was shown that 21-day-old pigs that were infected with the RH strain presented eye discharge and temperature elevation between 3-5 dpi (Bugni et al. 2008). In contrast, Moura et al. (2007) observed neither clinical nor haematological alterations in male adult pigs that were infected with oocysts (P strain) or tachyzoites (RH strain). Thus, the different clinical manifestations that were seen between these studies, including our observations, may be due to the breed, sex and age of the pigs and also the quantity, form and parasite strain that was used for the infections. It was reported that a pig that presented with neurological signs four days after oral infection with *T. gondii* oocysts died (Wingstrand et al. 1997). In our work, one of the RH group pigs presented aggressiveness during the chronic phase of the disease, which may probably be an indication of neurological alterations caused by the infection.

Significant haematological changes were only detected in the ME-49 group that presented an increase in band neutrophils. This clearly indicates that an immune response based on this cell was triggered after the infection. Because band neutrophils were only seen in the pigs that were orally infected (ME-49 strain), this response may probably be due to ileitis that increases recruitment of these cells in mice (Bliss et al. 2001) and in considerable amounts in the ileum itself (Dunay et al. 2008 ). Neutrophil infiltration during the oral toxoplasmosis acute phase in mice has been described and its depletion is lethal (Bliss et al. 2001); however, evidence was shown that neutrophils are not crucial for *T. gondii* control (Dunay et al. 2010). Nevertheless, these cells are important because they chemotactically attract dendritic cells that are essential to promote a T-helper 1 response (Bennouna et al. 2003). On the other hand, a neutropaenia tendency by 3 dpi was observed in *T. gondii* infected pigs; however, this study was carried out with very young pigs, as discussed above (Bugni et al. 2008), but with the same dose, parasite form and strain (RH) used in this work. Neutropaenia was observed during toxoplasmosis due to neutrophil migration to infected tissues (Dubey et al. 1994). The observed increase in band neutrophil counts was not sufficient to induce leucocytosis. It is known that only extensive tissue damage causes a significant increase of blood neutrophils that generates leucocytosis (Thrall et al. 2004). Thus, the ME-49 infection in the pigs may have caused enough tissue damage to increase band neutrophils in the blood, but it was not sufficient enough to induce leucocytosis.

Neutrophil changes were not seen in the RH group pigs, suggesting a lower immune response in comparison with the ME-49 group. Additionally, the infection venue may induce a completely distinct local response with different reflections in the systemic response, which is seen as leucocyte fluctuation in the peripheral blood.

The bioassay test done with blood leucocytes showed that the parasitaemia occurred only in the early days after infection of the pigs and the ME-49 strain presented a broader range. Nevertheless, tachyzoites were seen in blood smears in the early and late days of the infection. Thus, these strains probably gained blood circulation right after the infection and persisted in the pig circulation after considerable time without clinical signs or physiological alterations that were detected by routine haematological methods. Despite the low parasitaemia with the microscopic evaluation, *T. gondii* was more frequently observed in neutrophils, suggesting that this leucocyte can be important for parasitic dissemination in the pigs as demonstrated in mice (Norose et al. 2008, Coombes et al. 2013). The biological tests indicated that the ME-49 strain tachyzoites reached the blood faster, in higher amounts and for a longer period of time after infection by the oral route than that observed via intramuscular inoculation with the RH strain tachyzoites. This indicates that the former may be a better experimental model due to its faster dissemination. The data obtained with the RH group pigs were similar to a study that used the same strain that described positive biological assays between 3-47 dpi (Moura et al. 2007). It is commonly known that *T. gondii* can only be seen in the peripheral blood during the acute phase. In fact, most of the studies concerning *T. gondii* isolation in humans occurred in immunocompromised (Hofflin & Remington 1985) or immunocompetent patients who presented ocular toxoplasmosis (Amendoeira & Coutinho 1982). However, a recent study has broken this paradigm, because it showed that *T. gondii* could circulate in the blood of immunocompetent chronically infected humans (Silveira et al. 2011). Thus, the observed tachyzoites in the chronically infected pigs reported here was similar to what was reported in humans, indicating that the pig model may be a good model to understand chronic toxoplasmosis in humans.

The higher serum ALT levels in the RH group pigs may be a probable consequence of direct hepatic injury due to parasitic replication. The liver is an important organ for multiplication or defence against the RH strain, mainly until 14 dpi (Dubey et al. 1994). Lack of ALT changes in the ME-49 group pigs may suggest that this infection was well managed by the liver.

The ileum, mesenteric lymph nodes and heart were the only ME-49 infected pig tissues that induced serum conversion in the mice; however, with the bioassay, cysts were only observed in the brains of mice that were inoculated with heart tissue, suggesting a higher concentration of parasites in the heart tissue. These results are similar to those obtained in pigs that were infected with 10 or less VEG strain oocysts, which showed many cysts in the tongue, brain and heart (Dubey et al. 1996).

No morphological alteration was detected in the examined infected pig tissues, which was similar to results reported by Wingstrand et al. (1997). Kidney transplant may cause toxoplasmosis (Gharavi et al. 2011), indicating that this organ may harbour this parasite and, consequently, present morphological changes. By using mice, we recently observed nephron loss occurrences with a consequent kidney volume decrease, as well as light glomerular and tubular lesions (D Benchimol de Souza et al., unpublished observations). However, no such changes were observed in pigs in the present study. Lack of pathological changes in the tissues of the infected pigs reported here might be due to the period of time between the infections and analyses. Changes would probably be detected if the tissues were examined soon after the infection. Additionally, kidney morphological alterations and other haematological parameters, such as the monocytosis tendency seen at 7 dpi after infection with the ME-49 strain, would be better evaluated and understood if higher pig numbers were used for these experiments.

The BR-1 mini pigs that were infected with the ME-49 strain used in this work resulted in a suitable model for studying toxoplasmosis because these animals carried the infection, but survived and presented chronic toxoplasmosis, which is similar to the immunocompetence observed in humans. Additionally, *T. gondii* was able to infect these pigs with prolonged parasitaemia without clinical signs.
